# Mucoepidermoid Carcinoma Associated with Osteosarcoma in a True Malignant Mixed Tumor of the Submandibular Region

**DOI:** 10.1155/2015/694684

**Published:** 2015-10-27

**Authors:** Dario Marcotullio, Marco de Vincentiis, Giannicola Iannella, Bruna Cerbelli, Giuseppe Magliulo

**Affiliations:** ^1^Organi di Senso Department, University of “Sapienza”, Viale del Policlinico 151, 00161 Rome, Italy; ^2^Pathology Department, University of “Sapienza”, Viale Regina Elena 324, 00161 Rome, Italy

## Abstract

*Introduction*. True malignant mixed tumor, also known as carcinosarcoma, is a rare tumor of the salivary gland composed of both malignant epithelial and malignant mesenchymal elements. Frequently carcinosarcoma arises in the background of a preexisting pleomorphic adenoma; however, if no evidence of benign mixed tumor is present, the lesion is known as carcinosarcoma “de novo.” We reported the first case of true malignant mixed tumor of the submandibular gland composed of high grade mucoepidermoid carcinoma associated with osteosarcoma. *Case Presentation*. A 69-year-old Caucasian male came to our department complaining of the appearance of an asymptomatic left submandibular neoformation progressively increasing in size over 3 months. We opted for surgical treatment. Histological examination confirmed the diagnosis of carcinosarcoma with the coexistence of high grade mucoepidermoid carcinoma and osteosarcoma. *Conclusion*. To the best of our knowledge, in the true malignant mixed tumor of the submandibular gland, mucoepidermoid carcinoma associated with osteosarcoma has never been previously reported.

## 1. Introduction

True malignant mixed tumor, also known as carcinosarcoma, is an exceedingly rare tumor of the salivary gland composed of both malignant epithelial and malignant mesenchymal elements. Its incidence is comprised between 0.04% and 0.16% of all salivary gland tumors with the parotid gland being the most affected site [1–3].

The authors present a recent rare case of 69-year-old man with a malignant mixed tumor of the left submandibular gland consisting in the association of a mucoepidermoid carcinoma (MEC) and an osteosarcoma. To the best of our knowledge, in true malignant mixed tumor of the salivary gland, these microscopic findings have never been previously reported.

Clinical presentation and results of histological and immunohistochemical study are reported.

## 2. Case Presentation

A 69-year-old Caucasian male came to our department complaining of the appearance of an asymptomatic left submandibular neoformation progressively increasing in size over 3 months. Medical, family, and psychosocial history were negative for relevant information; also, no previous surgical treatments were reported.

On clinical examination the mass measured 8 × 5 cm arising from level Ib (submandibular region) and extended into levels III and IV. Such mass appeared adherent to the underlying structures with soft texture. No pain or other symptoms were present. Right cervical region, oropharynx, thyroid, and upper respiratory airways showed no involvement.

Ultrasound evaluation of the mass revealed a mixed tissue consistency of cystic and solid areas separated by high flow vascular fibrous septa in level Ib.

**Figure 1:**
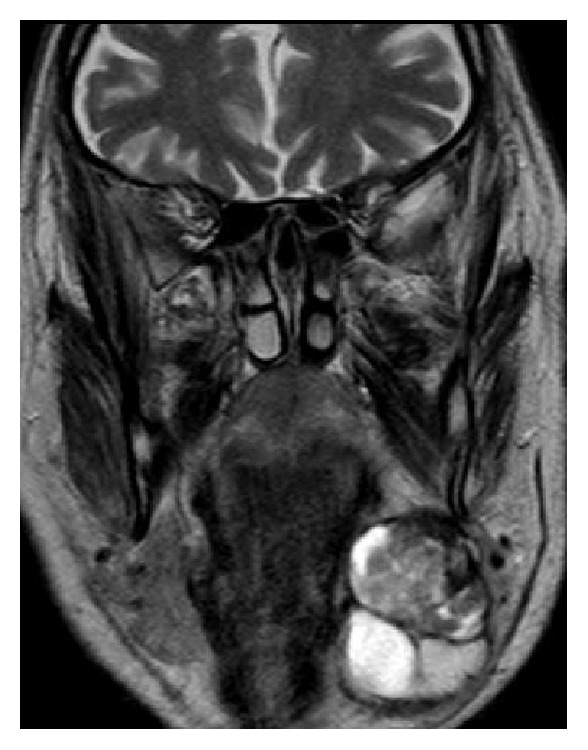
Preoperative MRI, coronal T2-W: 5 × 4 × 3 cm mass originating from the left submandibular gland. Two different multilobed neoformations with colliquate areas (black square and black trapezius) delimited by a thick pathological peripheral tissue are clearly visible.

Contrast-enhanced magnetic resonance imaging (MRI) of the head and neck (Figure 1) showed a 5 × 4 × 3 cm multilobed neoformation originating from the left submandibular gland. A fluid component and solid areas were inside visible. No distant metastases were detected.

To integrate MRI data, subsequent head and neck computed tomography (CT) was performed. This examination indicated colliquate areas delimited by a thick pathological peripheral tissue within the mass context.

First, fine-needle aspiration cytology specimen was performed to determine the nature of the disease. It showed malignant cells without distinctive features failing to identify the type of primary lesion. A new US-FNAC was subsequently executed; however, also in this case the primary type of lesion was not identified.

We opted for surgical treatment, completely removing the mass by means of ipsilateral neck dissection of Ib, IIa, III, and IV levels.

**Figure 2:**
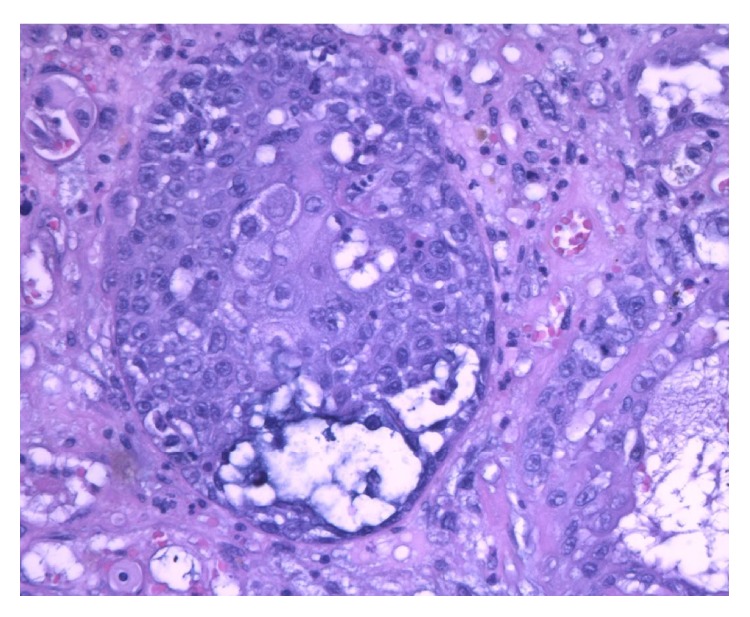
Mucoepidermoid carcinoma: glandular-like structure composed of a mixture of squamous and clear cells containing mucin (hematoxylin and eosin, 40x).

**Figure 3:**
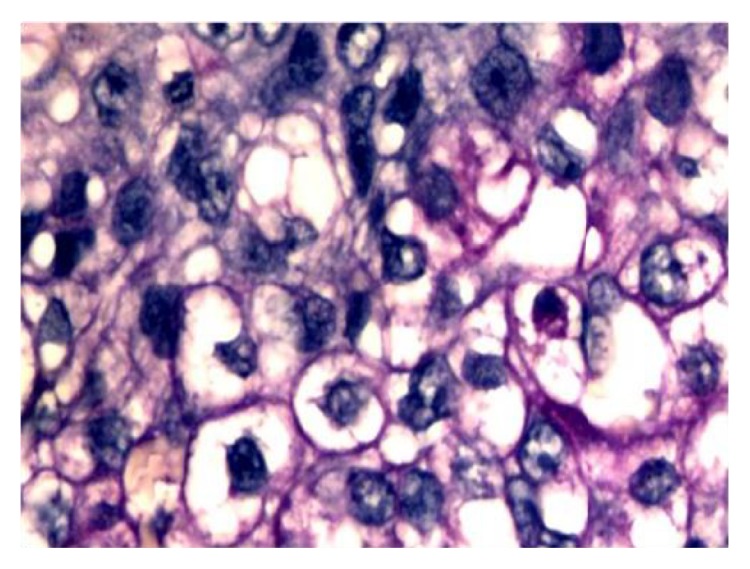
Some neoplastic epithelial cells with clear cytoplasm retain PAS positivity after diastase digestion (40x).

**Figure 4:**
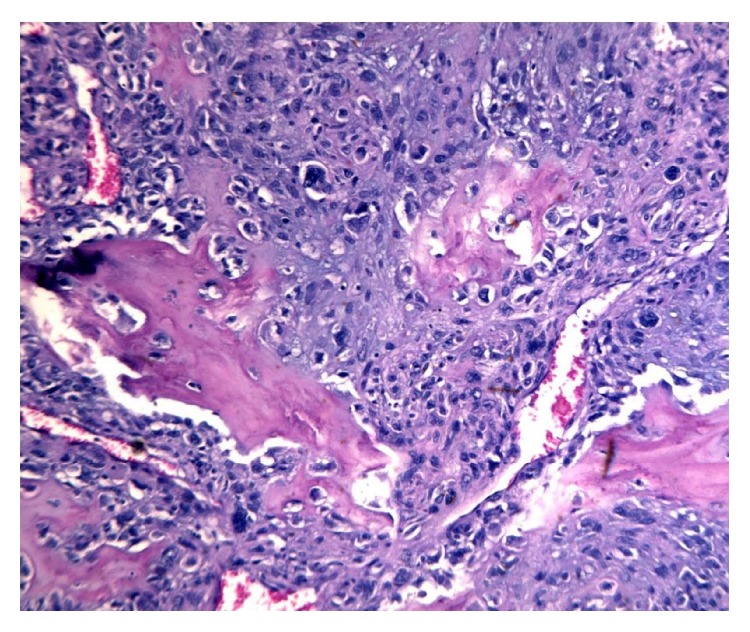
Osteosarcoma cells: mesenchymal elements of medium or large size with hyperchromatic, pleomorphic, and multinucleated nuclei arranged around an osteoid matrix (hematoxylin and eosin, 10x).

Histological examination showed a neoplastic proliferation characterized by two cellular components, substantially distinct from each other. No fusion of the different cell types could be seen. The first cellular component consisted of medium size elements with multilobed nucleus and slightly eosinophilic cytoplasm. Such elements appeared arranged in solid cell nests with central necrosis and cribriform areas (Figure 2). Cytokeratin and PAS positivity (Figure 3) were evident. The second cellular component consisted of mesenchymal elements of medium or large size with elongated hyperchromatic and pleomorphic nuclei. Such cellular elements appeared to be arranged around an eosinophilic material attributable to an osteoid matrix (Figure 4). Immunohistochemical study showed vimentin positivity. Tumor showed several cellular atypia, mitoses, areas of necrosis, or bleeding as well as angioinvasion.

Due to the coexistence of two separate cellular patterns, both of malignant nature, a diagnosis of carcinosarcoma, obtained by the fusion of high grade mucoepidermoid carcinoma and osteosarcoma, was made.

Postoperatively, the patient underwent intensity-modulated radiation with 66 Gray in 33 cycles.

At six-month follow-up no disease recurrence was revealed.

## 3. Discussion

True malignant mixed tumor (carcinosarcoma) of the salivary gland is an extremely rare tumor in which carcinomatous and sarcomatous components coexist and metastasize together [1–4].

Frequently, carcinosarcoma arises in the background of a preexisting pleomorphic adenoma and, in some cases, tumors were related to a previous history of radiotherapy. However, if none of these conditions is present, the lesion is classified as true malignant mixed tumor or carcinosarcoma “de novo” [3–5].  Table 1 shows the 31 established cases of carcinosarcoma “de novo” actually reported in the English literature. Stephen et al. [6] in 1986 published the largest series of true malignant mixed tumor with 12 cases of carcinosarcoma showed. Malignant epithelial component was ductal carcinoma in all patients, with 1 case of squamous component and 2 with undifferentiated features. About malignant mesenchymal elements 10 chondrosarcoma and 2 mixed malignant fibrous histiocytoma cases were reported.

The most common malignant epithelial components are squamous cell carcinoma or adenocarcinoma, whereas the malignant mesenchymal component mainly consists of chondrosarcoma, fibrosarcoma, or liposarcoma [1–5].

We reported the first case of true malignant mixed tumor of the salivary gland composed of high grade mucoepidermoid carcinoma and osteosarcoma.

Mucoepidermoid carcinoma is a common salivary tumor derived from ductal epithelium of the salivary gland, which displays a variety of biological behavior patterns. The high-grade variant is more aggressive with a poor prognosis, whereas the low-grade variant usually demonstrates satisfactory survival rates [7]. The diagnosis of MEC includes the identification of three intermixed tumor elements: mucin-producing cells, intermediate and/or clear cells, and squamoid cells [1, 7].

Osteosarcoma is the primary malignancy of bone with rare extraosseous head and neck localizations. Typical features of osteosarcoma are the presence of osteoid tissue within the neoformation, with extremely pleomorphic cells included in such osteoid matrix [8].

FNAC has a well-established role in the initial, preoperative diagnosis of salivary gland lesions. It is safe, fast, well tolerated, and minimally invasive; however, it is known to have several deficiencies. On average, FNAC has high specificity (97%), but the sensitivity is somewhat lower (80%). Thus, a positive diagnosis by FNAC is quite reliable, but the false-negative rate associated with FNAC (20%) may be unacceptable [9–12]. In addition, the fine-needle aspiration cytology is not considered effective for the diagnosis of true malignant mixed tumor [10–12]. In our case, FNAC showed malignant cells without distinctive features failing to identify the type of primary lesion.

Core needle biopsy (CNB) is a relatively new technique for the diagnosis of salivary gland masses that offers several potential advantages relative to FNAC [12, 13]. However, CNB was not performed in our patient.

Histological and immunohistochemical studies are essential both for a correct diagnosis and for distinguishing carcinosarcoma from other tumors. Usually, cytokeratin and epithelial membrane antigens are positive in the carcinomatous element while vimentin positivity is observed in the sarcomatous element [1–5].

No therapeutic protocol has been established for treating this atypical disease, because of limited individual or institutional experience. Treatment may consist of surgery alone or surgery and postoperative radiotherapy [1, 2, 14]. Staffieri et al. [14] compared the carcinosarcoma recurrence data in a group of patients who had undergone surgery versus surgery plus radiotherapy, with lower recurrence rate after the combination of surgery and radiotherapy (*p* = 0.3).

Due to the limited follow-up data reported in the literature, it is very difficult to comment specifically on tumor prognosis. Moreover, the different evolution of the disease could be explained by the histological subtypes observed. Considering 19 cases of de novo parotid carcinosarcoma with available data on follow-up, Staffieri et al. [14] observed that 31.6% of patients died after a median of 10.1 months from diagnosis. Taki et al. [5] reported a case report of carcinosarcoma consisting of chondrosarcoma and squamous cell carcinoma treated with total parotidectomy and radiation therapy without local or regionally recurrent disease after 14-month follow-up. In our case, after 6-month follow-up no disease recurrence was revealed. However, this time is not enough to consider a disease-free survival of our patient.

## 4. Conclusions

Salivary gland carcinosarcoma is a rare and highly aggressive disease with poor prognosis. The current treatment of choice is surgery followed by radiotherapy. However, long-term follow-up with patients who have already undergone treatment is necessary in further elucidating the clinical course of the disease. The association in a true malignant mixed tumor of mucoepidermoid carcinoma and osteosarcoma has never been reported previously, representing therefore a further possibility to be considered.

## Figures and Tables

**Table 1:** True mixed tumor: literature review.

Authors	Year	Number of cases	Site	Type of carcinoma	Type of sarcoma	Type of treatment	Follow-up	Recurrence
Clapp [15]	1966	1	Parotid gland	Adenocarcinoma	Angiosarcoma	Total Parotidectomy	—	—

King Jr. [16]	1967	1	Submandibular gland	Undifferentiated adenocarcinoma	Fibrosarcoma	Surgical resection and radiotherapy	1 year	No local recurrence or distant metastasis

Huntington and Dardick [17]	1985	1	Parotid gland	Ductal adenocarcinoma	Chondrosarcoma	Total parotidectomy	18 months	No local recurrence or distant metastasis

Stephen et al. [6]	1986	12	9: parotid gland 3: submandibular gland	All ductal carcinoma	10: chondrosarcoma 2: fibrosarcoma	Surgical resection in all cases	—	—

Dardick et al. [18]	1989	1	Parotid gland	Undifferentiated Adenocarcinoma	Chondrosarcoma	Total parotidectomy	—	—

Garner et al. [19]	1989	1	Parotid gland	Undifferentiated carcinoma	Chondrosarcoma/osteosarcoma	Total parotidectomy and adjuvant radiotherapy	18 months	No local recurrence or distant metastasis

Suzuki et al. [20]	1990	1	Submandibular gland	Undifferentiated carcinoma	Chondrosarcoma/osteosarcoma	Surgical resection and radiotherapy	1 year	No local recurrence or distant metastasis

Takata et al. [21]	1990	1	Tongue	Basaloid carcinoma	Chondrosarcoma/myxosarcoma/fibrosarcoma	Surgical resection and radiotherapy	—	—

Bleiweiss et al. [22]	1992	1	Submandibular gland	Adenocarcinoma	Chondrosarcoma/osteosarcoma	Surgical resection and radiotherapy	1 year	Local recurrence of the sarcomatous component

Lopez et al. [23]	1994	1	Parotid gland	Undifferentiated carcinoma	Chondrosarcoma	Total parotidectomy and adjuvant radiotherapy	13 months	No local recurrence or distant metastasis

Carson et al. [24]	1995	2	(i) Parotid gland	Both adenocarcinoma	(i) Chondrosarcoma/osteosarcoma	(i) Total parotidectomy + subsequent chemotherapy	(i) 9 months	(i) Died from local recurrence and aspiration pneumonia
			(ii) Submandibular gland		(ii) Leiomyosarcoma	(ii) Surgical resection	(ii) 9 months	(ii) No local recurrence or distant metastasis

Sironi et al. [9]	2000	1	Parotid gland	Squamous cell carcinoma	Osteosarcoma and myoepithelial malignant proliferation	Total parotidectomy and adjuvant radiotherapy	2 years	No local recurrence or distant metastasis

Kwon and Gu [1]	2001	1	Parotid gland	Squamous cell carcinoma	Rhabdomyosarcoma	Total parotidectomy and adjuvant radiotherapy	12 months	No local recurrence or distant metastasis

Pang et al. [2]	2001	1	Parotid gland	Squamous cell carcinoma	Chondrosarcoma	Total parotidectomy and right radical neck dissection	36 months	No local recurrence or distant metastasis

Mardi and Sharma [4]	2004	1	Parotid gland	Adenocarcinoma	Chondrosarcoma/osteosarcoma	Total parotidectomy	16 months	No local recurrence or distant metastasis

Staffieri et al. [14]	2007	1	Parotid gland	Adenocarcinoma	Chondrosarcoma/osteosarcoma	Surgical resection and adjuvant chemotherapy and radiotherapy	26-month follow-up	No local recurrence or distant metastasis

Morey-Mas et al. [25]	1997	1	Submandibular gland/salivary gland	Undifferentiated carcinoma	Chondrosarcoma	Surgical resection and radiotherapy	15 months	No local recurrence or distant metastasis

Tomas et al. [3]	2014	1	Parotid gland	Salivary duct adenocarcinoma	Malignant fibrous histiocytoma	Total parotidectomy and radiotherapy	9 months	No local recurrence or distant metastasis

Taki et al. [5]	2013	1	Parotid gland	Squamous cell carcinoma	Chondrosarcoma	Surgical resection and adjuvant radiotherapy	14 months	No local recurrence or distant metastasis
